# Characteristics of Fentanyl Overdose — Massachusetts, 2014–2016

**DOI:** 10.15585/mmwr.mm6614a2

**Published:** 2017-04-14

**Authors:** Nicholas J. Somerville, Julie O’Donnell, R. Matthew Gladden, Jon E. Zibbell, Traci C. Green, Morgan Younkin, Sarah Ruiz, Hermik Babakhanlou-Chase, Miranda Chan, Barry P. Callis, Janet Kuramoto-Crawford, Henry M. Nields, Alexander Y. Walley

**Affiliations:** ^1^Epidemic Intelligence Service, CDC; ^2^Massachusetts Department of Public Health; ^3^Substance Abuse and Mental Health Services Administration, Rockville, Maryland; ^4^Division of Unintentional Injury Prevention, National Center for Injury Prevention and Control, CDC; ^5^Boston Medical Center/Boston University School of Medicine, Boston, Massachusetts; ^6^Lawrence Family Medicine Residency, Lawrence, Massachusetts; ^7^Massachusetts Office of the Chief Medical Examiner.

Opioid overdose deaths in Massachusetts increased 150% from 2012 to 2015 (*1*). The proportion of opioid overdose deaths in the state involving fentanyl, a synthetic, short-acting opioid with 50–100 times the potency of morphine, increased from 32% during 2013–2014 to 74% in the first half of 2016 (*1*–*3*). In April 2015, the Drug Enforcement Agency (DEA) and CDC reported an increase in law enforcement fentanyl seizures in Massachusetts, much of which was believed to be illicitly manufactured fentanyl (IMF) (*4*). To guide overdose prevention and response activities, in April 2016, the Massachusetts Department of Public Health and the Office of the Chief Medical Examiner collaborated with CDC to investigate the characteristics of fentanyl overdose in three Massachusetts counties with high opioid overdose death rates. In these counties, medical examiner charts of opioid overdose decedents who died during October 1, 2014–March 31, 2015 were reviewed, and during April 2016, interviews were conducted with persons who used illicit opioids and witnessed or experienced an opioid overdose. Approximately two thirds of opioid overdose decedents tested positive for fentanyl on postmortem toxicology. Evidence for rapid progression of fentanyl overdose was common among both fatal and nonfatal overdoses. A majority of interview respondents reported successfully using multiple doses of naloxone, the antidote to opioid overdose, to reverse suspected fentanyl overdoses. Expanding and enhancing existing opioid overdose education and prevention programs to include fentanyl-specific messaging and practices could help public health authorities mitigate adverse effects associated with overdoses, especially in communities affected by IMF.

Barnstable, Bristol, and Plymouth counties in Massachusetts were investigated because of high opioid overdose death rates (estimated 29.8–34.5 per 100,000 population in 2015), and feasibility of interviewee recruitment through existing harm reduction programs in these counties (*5*).* To rapidly obtain a cross section of persons misusing opioids for semistructured, in-person interviews, a nonrandom sample of approximately 20 knowledgeable respondents per county was recruited with the help of harm reduction programs. Eligible persons were aged ≥18 years, lived in Massachusetts, had used illicit opioids during the previous 12 months, and had witnessed or experienced an opioid overdose during the previous 6 months. Equal numbers of men and women were recruited. Trained interviewers asked respondents about their experiences, knowledge, attitudes, and beliefs regarding opioid overdose. Interviews were audio recorded, transcribed, and thematically coded by multiple investigators.

Opioid overdose death data were abstracted from medical examiner charts, which included autopsy and toxicology reports, death scene reports, and emergency medical service logs. Abstracted charts met the following criteria: the death occurred during October 1, 2014–March 31, 2015; the decedent overdosed or resided in Barnstable, Bristol, or Plymouth counties; and opioids were listed as a contributing cause of death. Postmortem toxicology tests were used to categorize deaths as involving fentanyl (regardless of presence of other drugs), heroin or morphine (i.e., no fentanyl),^†^ or other opioids (e.g., prescription opioids). Fentanyl deaths were further categorized using death scene evidence as suspected IMF, suspected prescription fentanyl, or unknown source of fentanyl. Rapidity of overdose death was determined from available evidence, including needles inserted in decedents’ bodies, syringes found in hand, tourniquets still in place, and bystander reports of rapid unconsciousness after drug use. Demographic and overdose characteristic frequencies were examined by drug type.

Among 64 interview respondents, 52% were women, 61% were aged 25–44 years, and 81% were non-Hispanic white. Ninety-one percent reported that they were trained by a Massachusetts Department of Public Health-supported overdose education and naloxone distribution program in the use of naloxone for reversing an opioid overdose; trainees are taught that opioid overdose is defined by unresponsiveness and decreased respirations (*6*). During the 6 months before the interview, 95% of respondents witnessed an overdose and 42% overdosed themselves. Eighty-eight percent of respondents attributed the increase in opioid overdose deaths to suspected fentanyl, and 69% reported that suspected fentanyl was now available for purchase in powdered form (consistent with IMF preparation), and not as diverted prescription medications, (e.g., Duragesic transdermal fentanyl patch) (Box). Respondents reported that suspected fentanyl could be obtained alone or mixed with heroin, and persons using heroin often did not know whether fentanyl was mixed into the heroin they purchased. Respondents’ reactions to the addition of fentanyl to the illicit drug market varied. Although some persons sought out fentanyl and others attempted to avoid it, a majority of respondents reported that opioid-seeking behaviors were not altered in response to the emergence of fentanyl. A majority of respondents who witnessed a suspected fentanyl overdose (75%) described symptoms as occurring rapidly, within seconds to minutes. Twenty-five percent reported witnessing or experiencing an overdose when fentanyl was insufflated (snorted), and the remainder reported the overdose always involved injecting fentanyl. Atypical overdose characteristics described by respondents during suspected fentanyl overdose included immediate blue discoloration of the lips (20%), gurgling sounds with breathing (16%), stiffening of the body or seizure-like activity (13%), foaming at the mouth (6%), and confusion or strange affect before unresponsiveness (6%). Seventy-five percent of respondents reported witnessing naloxone administration, administering naloxone themselves, or receiving naloxone to successfully reverse an opioid or fentanyl overdose. Among these events, 83% of respondents reported that ≥2 naloxone doses (typical nasally administered dose in Massachusetts is 2 mg/2 mL^§^) per suspected fentanyl overdose were used before the person responded. Thirty percent of respondents reported using heroin or fentanyl with others present to help protect themselves from a fatal overdose.

BOX Sample quotations from persons who reported using opioids and who had witnessed or experienced an opioid overdose — Barnstable, Bristol, and Plymouth counties, Massachusetts, April 2016*

**Illicitly manufactured fentanyl (IMF) responsible for opioid overdose deaths**

“So, now what they [people selling illicit drugs] are doing is they’re cutting the heroin with the fentanyl to make it stronger. And the dope [heroin] is so strong with the fentanyl in it, that you get the whole dose of the fentanyl at once rather than being time-released [like the patch]. And that’s why people are dying—plain and simple. You know, they [people using illicit drugs] are doing the whole bag [of heroin mixed with fentanyl] and they don’t realize that they can’t handle it; their body can't handle it.”

**Overdoses involving IMF are acute and rapid**

“A person overdosing on regular dope [heroin] leans back and drops and then suddenly stops talking in a middle of a conversation and you look over and realize that they’re overdosing. Not like with fentanyl. I would say you notice it [a fentanyl overdose] as soon as they are done [injecting the fentanyl]. They don’t even have time to pull the needle out [of their body] and they’re on the ground.”

**Naloxone reverses overdoses involving IMF; multiple doses often required**

“So he put half [one dose] up one nose [nostril] and half [one dose] up the other nose, like they trained us to do, and she didn’t come to. So he put water on her face and kind of slapped her, which doesn’t really make you come to [regain consciousness]. It doesn’t. So he pulled out another thing of Narcan [brand of naloxone] and he put half of it [another dose] up one nose and then she came to…She just didn’t remember anything. She said, ‘What happened? I remember washing my hands and, like, what happened?’ We said, ‘You just overdosed in this room!’ So yeah, it was wicked scary.”

**Self-protective measures often employed**

“Like I will do a very, very, very little bit of fentanyl…and if I don’t feel it, I will do that little bit plus half. I’m just not going to throw the whole thing in the cooker and then do it, no way. I just know better.”

**Co-use of opioids and benzodiazepines**

“My daughter’s mother had benzos. And when she did one bag of heroin she already had done four or five Klonopin [brand of clonazepam] and she just died. That was it. She went into a coma for the night and she was dead in the morning.”

* Categories are not mutually exclusive; all respondents reported using opioids in the past 12 months and had witnessed or experienced an overdose, or both.

Among 196 opioid overdose decedents whose records were reviewed, 73% were men, 50% were aged 15–34 years, and 91% were non-Hispanic white. Demographics of fentanyl overdose decedents were similar to those of the overall opioid overdose decedents (Table). Among all opioid overdose decedents 64% tested positive for fentanyl on postmortem toxicology; this proportion increased from 44% in October 2014 to 76% in March 2015 (Figure). Eighty-two percent of fentanyl deaths were suspected to involve IMF, 4% were suspected to involve prescription fentanyl, and 14% involved an unknown source of fentanyl. Thirty-six percent of fentanyl deaths had evidence of an overdose occurring within seconds to minutes after drug use, and 90% of fentanyl overdose decedents were pulseless upon emergency medical services arrival (Table). Ninety-one percent of fatal fentanyl overdoses occurred in a hotel, motel, or private residence. Only 6% of fentanyl overdose deaths had evidence of lay bystander-administered naloxone, which is available from pharmacies and harm reduction programs in Massachusetts. In addition to the limited use of naloxone by laypersons, rapid bystander response to fentanyl overdose was inhibited by lack of bystanders (18%), spatial separation of decedents from bystanders (e.g., person was in another room of the house [58%]), lack of awareness of decedent’s drug use by bystanders (24%), intoxication of bystanders who were present (12%), failure of bystanders to recognize overdose symptoms (11%), or bystander assumption that the decedent had gone to sleep (15%). Clear evidence that a bystander was unimpaired, witnessed the drug consumption, and was present during an overdose (i.e., able to respond immediately) was reported in 1% of the fentanyl overdose decedent charts.

**TABLE:** Demographic characteristics and overdose precipitating circumstances of fentanyl overdose decedents (N = 125) — Barnstable, Bristol, and Plymouth counties, Massachusetts, October 1, 2014–March 31, 2015

Characteristic	No. (%)
**Sex**	
Male	100 (80)
Female	25 (20)
**Age group (yrs)**	
15–24	15 (12)
25–34	52 (42)
35–44	24 (19)
≥45	34 (27)
**Race/Ethnicity**	
White, non-Hispanic	111 (89)
Other	14 (11)
**Location of overdose**	
Decedent's home	85 (68)
Other private residence	22 (18)
Hotel or motel	7 (6)
Other	11 (9)
**Overdose onset, pulselessness, and bystander naloxone administration**	
Evidence of rapid onset of overdose symptoms	45 (36)
Pulseless upon emergency medical services arrival	112 (90)
Evidence of bystander naloxone administration	7 (6)
**Barriers to bystander response**	
No bystander present	23 (18)
Decedent spatially separated from any bystander*	73 (58)
Bystander unaware of decedent’s drug use	30 (24)
Bystander also using drugs or alcohol	15 (12)
Bystander reported symptoms of intoxication or overdose (snoring, falling asleep, or nodding), but did not realize decedent was overdosing	14 (11)
Decedent was thought to have gone to sleep	19 (15)
**Route of drug administration^†^**	
Evidence of injection	83 (66)
Evidence of insufflation (snorting)	11 (9)
No evidence of route of administration	26 (21)

* Spatial separation defined as having a bystander nearby, either during or shortly preceding the overdose, who potentially had an opportunity to intervene and respond to the overdose, but who was not in the same room or physical space as the decedent.

^†^ Categories were not defined as mutually exclusive, but all records with evidence of injection had no evidence of insufflation, and all records with evidence of insufflation had no evidence of injection. Any evidence of route of administration was coded but not linked to specific drugs.

**FIGURE:**
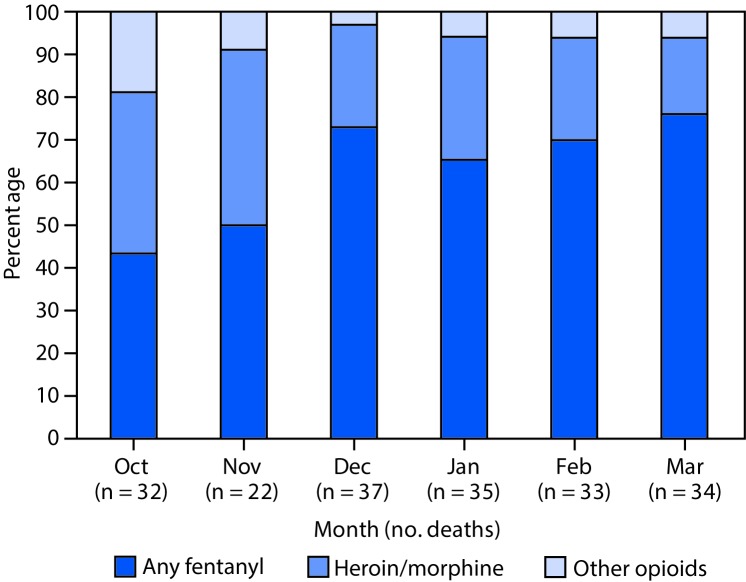
Percentage of opioid overdose deaths involving fentanyl, heroin/morphine (without fentanyl), and other opioids (without fentanyl, heroin/morphine) — Barnstable, Bristol, and Plymouth counties, Massachusetts, October 2014–March 2015

## Discussion

Introduction of fentanyl into the illicit drug market has been a major contributing factor to the rapid increase in opioid overdoses in southeastern Massachusetts and reflects a growing national public health issue (*7*). Previous DEA reports (*4*) and the findings of this investigation indicate that IMF is widely available through illicit drug markets in southeastern Massachusetts, and that the majority of fentanyl linked to fatal overdoses is suspected IMF rather than diverted prescription fentanyl. Taken together, these data highlight the need to integrate fentanyl testing into standard substance use toxicology tests employed by the medical, criminal justice, and treatment communities in Massachusetts areas with high levels of fentanyl use and overdose.

Evidence from over one third of medical examiner charts and reports from 75% of interview respondents demonstrated that fentanyl overdose can begin suddenly, progress to death rapidly, and manifest atypical physical symptoms. Timely administration of a sufficient naloxone dose by a trained layperson or emergency medical services responder can reverse fentanyl overdose. Although bystanders were frequently present in the general location of overdose death, timely bystander naloxone administration did not occur because bystanders did not have naloxone, were spatially separated or impaired by substance use, or failed to recognize overdose symptoms. Findings indicate that persons using fentanyl have an increased chance of surviving an overdose if directly observed by someone trained and equipped with sufficient doses of naloxone. In some countries, including Canada and Australia, overdose morbidity and mortality rates have decreased in areas near supervised injection facilities where personnel are available to observe overdose onset, if it occurs, and administer naloxone as needed (*8*). Because multiple doses might be required to reverse a fentanyl overdose, emergency medical services and community naloxone distribution programs might need to ensure that appropriate numbers of doses are distributed.

The findings in this report are subject to at least three limitations. First, toxicology reports in medical examiner charts cannot distinguish between prescription fentanyl and IMF; therefore, categorization was completed using death scene evidence, which varied and sometimes was inconclusive. In addition, samples were not tested for emerging fentanyl analogs, such as carfentanil. Overdose deaths were also categorized broadly as involving fentanyl, heroin or morphine, or other opioids, although in many cases other drugs also contributed to the death. Atypical symptoms reported during fentanyl overdose may be attributable to other drugs or drug combinations and not fentanyl. Second, circumstances or events preceding death (e.g., rapid onset of overdose symptoms) can be inferred from death scene evidence, but absence of evidence cannot be interpreted as evidence of absence; numbers presented therefore likely underestimate the actual prevalence of circumstances. Finally, interview respondents were recruited with the help of community-based harm reduction programs in which overdose prevention education and naloxone rescue kits were offered. Thus, this sample population was potentially more informed about and experienced with fentanyl, naloxone, overdose prevention and treatment, and rescue efforts than are all persons who use illicit opioids. In addition, interview comparability is limited because not all respondents were asked uniform questions.

Adaptation of harm reduction practices designed to reduce health-related consequences of unsafe drug use, including the addition of warnings about fentanyl’s characteristics and toxicity, could mitigate the fentanyl-related impact of the U.S. opioid epidemic in communities affected by fentanyl. Population-based strategies to prevent and reduce opioid use and opioid use disorders, such as expansion of access to evidence-based treatment, are likely to be effective in preventing fentanyl overdose and death. The high percentage of fatal overdoses occurring at home with no naloxone present, coupled with the rapid onset of overdose symptoms after using fentanyl through injection or insufflation, underscores the urgent need to expand initiatives to link persons at high risk for overdose (such as persons using heroin, persons with past overdoses, or persons recently released from incarceration) to harm reduction services and evidence-based treatment (*2*,*8*).

**Summary**

**What is already known about this topic?**

Fentanyl has a growing presence in the illicit drug market and is involved in an increasing proportion of opioid overdose deaths.

**What is added by this report?**

Approximately two thirds of investigated opioid overdose deaths in southeastern Massachusetts during October 1, 2014–March 31, 2015 involved fentanyl, a majority of which was suspected illicitly manufactured fentanyl (IMF), reported to be widely available in the illicit drug market. Fentanyl overdose can progress rapidly, and a majority of decedents were physically separated from bystanders. Naloxone can reverse fentanyl overdose if administered in sufficient dosage immediately upon recognition of overdose symptoms.

**What are the implications for public health practice?**

A comprehensive public health response is needed to address overdoses related to IMF. First, fentanyl should be included on standard toxicology screens to facilitate early identification. Second, existing harm reduction strategies to identify likely fentanyl exposure should be adapted, such as training for bystanders that includes direct observation of anyone injecting or insufflating illicit opioids, ensuring that trained bystanders are equipped with sufficient doses of naloxone, expanding layperson training, and providing access to naloxone. Third, access and linkages to medication for opioid use disorders need to be enhanced in fentanyl-affected areas.

## Footnotes

* County-specific rates were calculated using the number of deaths listed in this reference and population counts from CDC WONDER (https://wonder.cdc.gov/).

† There is no toxicology test specifically for heroin. Heroin metabolizes into 6‐monoacetylmorphine (6-MAM), which in turn is rapidly metabolized to morphine. Deaths in which the decedent tested positive for 6-MAM or morphine were classified as heroin- or morphine-involved. Recent evidence from Massachusetts indicates that a majority of morphine-positive toxicology test results are due to heroin metabolism. http://www.mass.gov/eohhs/gov/departments/dph/stop-addiction/chapter-55-overdose-assessment.html.

§
http://prescribetoprevent.org/pharmacists/formulations .

## References

[1] Massachusetts Department of Public Health. Data brief: opioid-related overdose deaths among Massachusetts residents. Boston, MA: Commonwealth of Massachusetts, Executive Office of Health and Human Services, Department of Public Health; 2016. http://www.mass.gov/eohhs/docs/dph/stop-addiction/current-statistics/data-brief-overdose-deaths-nov-2016-ma-residents.pdf

[2] Massachusetts Department of Public Health. An assessment of opioid-related deaths in Massachusetts (2013–2014). Boston, MA: Commonwealth of Massachusetts, Executive Office of Health and Human Services, Department of Public Health; 2016. http://www.mass.gov/eohhs/gov/departments/dph/stop-addiction/chapter-55-overdose-assessment.html

[3] Algren DA, Monteilh CP, Punja M, Fentanyl-associated fatalities among illicit drug users in Wayne County, Michigan (July 2005–May 2006). J Med Toxicol 2013;9:106–15. 10.1007/s13181-012-0285 23359211 PMC3576499

[4] CDC. Increases in fentanyl drug confiscations and fentanyl-related overdose fatalities. Atlanta, GA: US Department of Health and Human Services, CDC; 2015. https://emergency.cdc.gov/han/han00384.asp

[5] Massachusetts Department of Public Health. Number of unintentional opioid-related overdose deaths by county, MA residents: 2000–2015. Boston, MA: Commonwealth of Massachusetts, Executive Office of Health and Human Services, Department of Public Health; 2016. http://www.mass.gov/eohhs/docs/dph/stop-addiction/current-statistics/overdose-deaths-by-county-nov-2016.pdf

[6] Walley AY, Xuan Z, Hackman HH, Opioid overdose rates and implementation of overdose education and nasal naloxone distribution in Massachusetts: interrupted time series analysis. BMJ 2013;346(jan30 5):f174. 10.1136/bmj.f174 PMC4688551 23372174

[7] Rudd RA, Seth P, David F, Scholl L. Increases in drug and opioid-involved overdose deaths—United States, 2010–2015. MMWR Morb Mortal Wkly Rep 2016;65:1445–52. 10.15585/mmwr.mm655051e1 28033313

[8] Potier C, Laprévote V, Dubois-Arber F, Cottencin O, Rolland B. Supervised injection services: what has been demonstrated? A systematic literature review. Drug Alcohol Depend 2014;145:48–68. 10.1016/j.drugalcdep.2014.10.012 25456324

